# Supplementary material for: Polychromatic solar energy conversion in pigment-protein chimeras that unite the two kingdoms of (bacterio)chlorophyll-based photosynthesis
Source: Nat Commun. 2020 Mar 24;11:1542. doi: 10.1038/s41467-020-15321-w (PMC7093453; doi:10.1038/s41467-020-15321-w)
Supplement: Supplementary file 2 — Peer Review File [file 41467_2020_15321_MOESM2_ESM.pdf]

Editorial Note: This manuscript has been previously reviewed at another journal that is not operating a transparent peer review scheme. This document only contains reviewer comments and rebuttal letters for versions considered at Nature Communications.

REVIEWERS' COMMENTS:

Reviewer #1 (Remarks to the Author):

I thank the authors for addressing my issues.

Reviewer #2 (Remarks to the Author):

Now, I feel that my questions and concerns have been properly addressed. The revised manuscript has been improved in both clarity and completeness.

Response to reviewer comments.

REVIEWERS' COMMENTS:
Reviewer #1 (Remarks to the Author):

I thank the authors for addressing my issues.

Reviewer #2 (Remarks to the Author):

Now, I feel that my questions and concerns have been properly addressed. The revised manuscript has been improved in both clarity and completeness.

We are pleased that the changes we made to the manuscript have satisfied the comments and suggestions of the reviewers.
